# Opinion: Commensal papillomavirus immunity preserves the homeostasis of highly mutated normal skin

**DOI:** 10.3389/fmicb.2025.1576308

**Published:** 2025-05-05

**Authors:** Nicholas A. Wallace, Baki Akgül

**Affiliations:** ^1^Department of Kinesiology, Kansas State University, Manhattan, KS, United States; ^2^Institute of Virology, National Reference Center for Papilloma- and Polyomaviruses, University of Cologne, Faculty of Medicine and University Hospital of Cologne, Cologne, Germany

**Keywords:** human papillomavirus (HPV), beta HPV, skin homeostasis, skin carcinogenesis, skin cancer mouse model

## Introduction

The relationship between human papillomavirus (HPV) of genus betapapillomavirus (β-HPV) and skin carcinogenesis has long been a subject of intrigue. While high-risk HPVs of the genus Alphapapillomavirus (α-HPV) are known to cause anogenital and oropharyngeal cancers, the tumorigenic potential of β-HPVs is less clear. For decades, a subset of β-HPVs has been postulated to promote cutaneous squamous cell cancer (cSCC) via a “hit-and-run” mechanism, in which infections act transiently during early oncogenesis (Hasche et al., 2018). This hypothesis is based on the observation that viral loads in precancerous actinic keratoses are significantly higher than those in established cancers (Weissenborn et al., 2005; Dona et al., 2019), and that gene products from some β-HPVs promote ultraviolet (UV) light-induced genetic mutations (Wendel and Wallace, 2017). This suggests a strong negative selection against β-HPVs, yet serology studies suggest that most people are regularly infected with β-HPVs.

From an evolutionary perspective, the long-standing relationship between β-HPVs and humans suggests that viruses have adapted to coexist with and even benefit their hosts. In a recent publication by Son et al. (2024), the Demehri group provides compelling evidence supporting an intricate role of β-HPV in the homeostasis of UV-damaged skin. Their data suggest that β-HPV infection can protect against oncogenesis by acting as both a marker and a regulator of early oncogenic events. Using the mouse papillomavirus (MMuPV1) model, they show that immunity to cutaneous papillomaviruses limits the expansion of mutant p53 keratinocyte clones, induced by extensive UV exposure. Virus-specific CD8+ T cells are critical for maintaining skin integrity by targeting the mutated cells. The loss of functional p53 appears to unmask β-HPV antigens and tag affected cells for immune-mediated clearance. Interestingly, the expansion of p53-mutant clones in premalignant lesions (actinic keratoses) was inversely correlated with the presence of β-HPV, suggesting that the virus “flags” mutated cells for immune surveillance and that eliminating β-HPV infections promotes lesion progression.

This study's insights go beyond providing a nuanced interplay between virome and host immunity in cancer prevention, suggesting that enhancing β-HPV-specific T cell immunity could represent a promising avenue for cSCC prevention. Additionally, the results challenge the traditional view of eukaryotic DNA viruses as merely latent passengers in immunocompetent hosts, casting them instead as active regulators of tissue homeostasis. This work highlights an exciting evolutionary symbiosis wherein commensal viruses appear to have evolved with their hosts to support tissue integrity in the face of a mutational burden.

Despite these interesting findings, there are knowledge gaps that have not been well addressed. Most strikingly, studies on MmuPV1 have found that utilizing immunosuppressive doses of UV light the virus promotes, rather than prevents, tumorigenesis (Uberoi et al., 2016; Spurgeon and Lambert, 2020). Understanding how the same cutaneous viral infection could have divergent effects when different UV doses are applied is a critical next step before laboratory findings can guide clinical interventions. Furthermore, Son et al. did not address the heterogeneity among cutaneous HPVs, a diverse group with varying degrees of similarity to MMuPV1, and distinct associations with disease, particularly in immunosuppressed individuals. It is plausible that not all cutaneous HPVs provide equal protection against skin cancer. Indeed, one notable difference between MMuPV1 and the β-HPVs most closely connected with skin cancer development (β-HPV types HPV5 and HPV8) is the ability of these viruses to disrupt DNA repair. The E6 gene product from β-HPV types HPV5 and HPV8 promotes mutations in the host genome by binding and destabilizing a histone acetyltransferase, p300 (Hufbauer et al., 2015; Wendel and Wallace, 2017; Dacus and Wallace, 2021). In contrast, MMuPV1 E6 neither binds nor destabilizes p300 (Meyers et al., 2017). Some, but not all, β-HPV E6 proteins are predicted to bind to p300, suggesting that the findings reported by Son et al. could be most relevant to β-HPVs with E6 proteins that do not bind to p300. Further exploration of the diversity of the β*-HPV* genus is crucial for leveraging clinical insights. We look forward to follow-up studies that delve deeper into the molecular triggers of β-HPV loss during carcinogenesis and explore the therapeutic potential of modulating β-HPV-related immune responses.

## Discussion

Therapies enhancing β-HPV-specific T cell immunity have great promise but must first address variations in the immune response due to immunosuppression or viral load. Given that cSCC constitutes a significant and rising economic burden, understanding and leveraging β-HPV-specific immunity could pave the way for transformative strategies for both the prevention and management of skin cancer.

## Data Availability

The original contributions presented in the study are included in the article/supplementary material, further inquiries can be directed to the corresponding author.
